# Supplementary material for: Prognostication of DNA Damage Response Protein Expression Patterns in Chronic Lymphocytic Leukemia
Source: Int J Mol Sci. 2023 Mar 13;24(6):5481. doi: 10.3390/ijms24065481 (PMC10049670; doi:10.3390/ijms24065481)
Supplement: Supplementary file 1 [file ijms-24-05481-s001.zip › ijms-2237716-supplementary.pdf]

	TOTAL		C1	C2	C3	*P*-value
**Number**	**795**		**256**	**51**	**488**	
**Age (Mean +/- STD)**		65 (± 9.8)	65 (± 11)	65 (± 11)	65 ± 11	0.91
**Vital Status (Dead)**	88	11.1%	14.8%	17.6%	8.4%	**0.02**
**Race**	**Number**	**Percentage**				0.97
Asian	7	0.9%	0.4%	2.1%	1.1%	
Black	33	4.2%	5.2%	2.1%	4.0%	
Hispanic	22	2.9%	2.8%	2.1%	2.9%	
White	710	92.0%	91.5%	93.6%	92.0%	
**Gender**						0.12
Female	310	39.0%	35.2%	52.9%	39.5%	
Male	485	61.0%	64.8%	47.1%	60.5%	
**Binet Stage**						0.54
A	478	478 (61.0%)	59.5%	29 (58.0%)	62.0%	
B	71	71 (9.06%)	7.9%	2 (4.00%)	10.2%	
C	235	235 (30.0%)	32.5%	19 (38.0%)	27.8%	
**Rai Stage**						0.61
0	268	34.2%	34.5%	38.0%	33.6%	
I	234	29.8%	27.8%	22.0%	31.7%	
II	47	6.0%	5.1%	2.0%	6.9%	
III	132	16.8%	17.9%	28.0%	15.1%	
IV	103	13.1%	14.7%	10.0%	12.7%	
**Biomarkers**						
**IGHV Status (Unmutated)**	**280**	48.6%	58.3%	25.0%	45.7%	**0.002**
**ZAP70**	**189**	50.3%	59.3%	40.5%	47.1%	0.10
**SF3B1**	**34**	16.1%	19.8%	14.3%	13.8%	0.73
**Cytogenetic abberations**						
**Deletion 11Q**	**100**	14.1%	19.0%	6.5%	12.2%	0.05
**Deletion 13Q**	**273**	38.4%	22.0%	37.0%	47.3%	**<0.001**
**Trisomy 12**	**109**	15.3%	30.0%	17.4%	7.2%	**<0.001**
**Deletion 17P**	**68**	95.6%	10.8%	13.0%	8.6%	0.67
**TP53**	**34**	4.3%	4.3%	2.0%	4.5%	0.866
**No Abberations**	**165**	23.2%	20.7%	28.3%	24.0%	0.65
**Lab Tests**	**Units**					
**PB Platelets**	**K/uL**	190 (± 72)	190 (± 75)	220 (± 77)	190 (± 70)	**0.03**

**Hemoglobin**	**g/dL**	13 (± 1.8)	13 (± 2.0)	14 (± 1.5)	14 (± 1.7)	0.90
**Serum B2M**	**mg/L**	2.8 (± 1.8)	2.7 (± 1.4)	2.2 (± 1.0)	2.8 (± 2.0)	0.07
**Serum LDH**	**IU**	480 (± 240)	490 (± 300)	520 (± 210)	460 (± 200)	0.27
**Lymphocytes**	**K/uL**	38 (± 54)	42 (± 61)	18 (± 19)	38 (± 51)	**0.02**
**Immunophenotypic Markers**						
**CD5**	**% cells positive+**	94 (± 11)	93 (± 9.5)	94 (± 4.0)	94 (± 12)	0.15
**CD19**		81 (± 15)	82 (± 16)	76 (± 16)	82 (± 14)	0.09
**CD20**		78 (± 20)	78 (± 21)	79 (± 22)	78 (± 19)	0.69
**CD22**		63 (± 39)	68 (± 38)	74 (± 37)	59 (± 40)	**<0.001**
**CD23**		87 (± 18)	86 (± 19)	85 (± 20)	88 (± 17)	0.99
**CD38**		24 (± 27)	33 (± 31)	23 (± 29)	19 (± 23)	**<0.001**
**CD79b**		43 (± 38)	48 (± 33)	40 (± 36)	40 (± 40)	**0.02**

Table 2

	Univariate Overall Survival				Multivariate Overall Survival			
**Variable**	**Est.**	**2.50%**	**97.50%**	***p*-value**	**Est.**	**2.50%**	**97.50 %**	***p*-value**
DDR Cluster 1	6.53	5.82	7.24	*P* <0.01	8.32	4.01	12.62	***P* <0.01**
DDR Cluster 2	6.37	4.77	7.96	*P* <0.01	7.04	2.39	11.7	***P* <0.01**
DDR Cluster 3	7.73	7.22	8.25	*P* <0.01	9.3	5.28	13.32	***P* <0.01**
Gender Male	6.98	6.46	7.5	*P* <0.01	-0.69	-2.07	0.69	0.33
Binet Stage B	9.62	8.28	10.96	*P* <0.01	2.17	-0.72	5.05	0.14
Binet Stage C	5.97	5.23	6.71	*P* <0.01	-0.52	-3.59	2.55	0.74
Rai Stage I	8.91	8.17	9.64	*P* <0.01	1.7	-0.2	3.59	0.08
Rai Stage II	8.49	6.85	10.13	*P* <0.01	1.46	-1.98	4.89	0.4
Rai Stage III	5.58	4.6	6.56	*P* <0.01	-1.01	-2.83	0.81	0.27
Rai Stage IV	6.47	5.37	7.58	*P* <0.01	0.01	-1.83	1.86	0.99
Del_11QPOS	6.47	5.38	7.57	*P* <0.01	-1.43	-5.2	2.34	0.46
Del_13QPOS	6.79	6.13	7.46	*P* <0.01	-1.47	-5.18	2.23	0.44
Del_17PPOS	7.41	6.08	8.74	*P* <0.01	-0.52	-4.44	3.41	0.8
T12POS	5.67	4.63	6.71	*P* <0.01	-2	-5.78	1.79	0.3
No Major Mutation	6.58	6.12	7.05	*P* <0.01	-0.9	-4.79	3	0.65
IGHV Unmutated	6.32	5.66	6.99	*P* <0.01	0.31	-1.18	1.79	0.68
Zap70POS	6.28	5.53	7.03	*P* <0.01	-1.56	-2.99	-0.13	**0.03**

Binet Stage A and Rai Stage 0 used as comparator
